# Early-Life Stress Induced Epigenetic Changes of Corticotropin-Releasing Factor Gene in Anorexic Low Body Weight–Selected Chicks

**DOI:** 10.3390/life10050051

**Published:** 2020-04-27

**Authors:** Yang Xiao, Jinxin Wang, Paul B. Siegel, Mark A. Cline, Elizabeth R. Gilbert

**Affiliations:** 1 Department of Animal and Poultry Sciences, Virginia Polytechnic Institute and State University, Blacksburg, VA 24061, USA; xyang91@vt.edu (Y.X.); jxwang59@vt.edu (J.W.); pbsiegel@vt.edu (P.B.S.); macline2@vt.edu (M.A.C.); 2 School of Neuroscience, Virginia Polytechnic Institute and State University, Blacksburg, VA 24061, USA

**Keywords:** anorexia, ARC, chicks, CRF, DNA methylation, early-life stressor, hypothalamus, MBD2, NPY, PVN

## Abstract

The expression of neuropeptide Y (*NPY*) in the arcuate nucleus (ARC) and corticotropin-releasing factor (*CRF*) in the paraventricular nucleus (PVN) were increased when low body weight–selected (LWS) line chicks, which are predisposed to anorexia, were subjected to a combination of nutritional and thermal stressors at hatch. We hypothesized that such changes resulted from epigenetic modifications. We determined global DNA methylation, DNA methyltransferase (DNMT) activity, and methylation near the promoter regions of *NPY* and *CRF*, in the hypothalamus of LWS chicks on day 5 post-hatch. Stress exposure at hatch induced global hypermethylation and increased DNMT activity in the ARC but not PVN. In the PVN of stressed LWS chicks, there was decreased methylation of a CpG site located at the core binding domain of methyl cytosine binding domain protein 2 (MBD2), near the *CRF* gene promoter. We then demonstrated that this was associated with disrupted binding of MBD2. There was also reduced utilization of yolk reserves and lean and fat masses in chicks that were stress-exposed. These findings provide novel insights on molecular mechanisms through which stressful events induce or intensify anorexia in predisposed individuals and a novel molecular target for further studies.

## 1. Introduction

Appetite regulatory signals, stress responses, and thermoregulation are integrated at the level of the hypothalamus. Although environmental stressors can alter feeding behavior, the molecular mechanisms are unclear and effects that persist with age likely have an epigenetic underpinning. A model that can be used to study these mechanisms are the Virginia body weight lines of chickens. Selection for low or high juvenile body weight for more than 60 consecutive generations has resulted in correlated responses in appetite regulation, body composition, and stress susceptibility [1,2]. One of the most striking differences in appetite regulation in the lines is with respect to the orexigenic effects of neuropeptide Y (*NPY*). Under some conditions, the low body weight–selected (LWS) chicks are completely refractory to the food intake–stimulating effects of centrally administered *NPY* [3]. Specifically, this change in appetite regulation is stress-induced, as a combination of transient low temperature coupled to delayed access to food at hatch rendered the LWS but not high body weight–selected (HWS) chicks resistant to the hunger-promoting effects of *NPY*. The LWS chicks hatched and reared under thermoneutral conditions with full access to food responded to *NPY* with increased food intake albeit not as robustly as HWS chicks, which responded vigorously under all conditions. [4,5]. *NPY* is one of the most potent orexigenic factors in mammals and birds [6], and these results suggest that increased anorexigenic tone overrides the effects of *NPY*, which is quite remarkable given the potency of NPY at stimulating food intake.

At the molecular level, there was increased mRNA expression of *NPY* in the arcuate nucleus (ARC) and corticotropin-releasing factor (*CRF*) in the paraventricular nucleus (PVN) of the hypothalamus in stressed LWS but not HWS chicks at 5 days post-hatch [4]. The ARC is vital for appetite regulation and energy homeostasis [7]. Both orexigenic *NPY* and agouti-related peptide (AgRP) neurons and anorexigenic pro-opiomelanocortin and cocaine- and amphetamine-regulated transcript neurons are found in the ARC, which direct axons to second-order nuclei like the PVN [8]. The PVN suppresses appetite and it is the major source of *CRF*, which inhibits feeding behavior and is part of the stress response [9]. In addition to up-regulated *CRF* mRNA in the PVN, stressed LWS chicks also had increased circulating corticosterone [4,5]. The reversal of resistance to *NPY* by injecting a CRF receptor antagonist prior to exposure to the stressors verified that CRF signaling in the PVN may override the orexigenic effect of *NPY* and exacerbate anorexia in LWS chicks [4].

Although the research presented here with the LWS line does not include changes that persist post-sexual maturity, there is a growing body of evidence showing that stress at young ages can shape the development of an organism and lead to long-term physiological alterations, such as changing body composition and nutrient metabolism [10]. Thus, the organism is pre-disposed to chronic metabolic and mental disorders [11,12,13,14]. When an animal is stressed, CRF is secreted from the PVN to stimulate the release of adrenocorticotropic hormone (ACTH) from the anterior pituitary [15]. ACTH then acts on the adrenal cortex to regulate production of glucocorticoids, which provide negative feedback by suppressing CRF to maintain homeostasis [16]. However, exposure to stress early in life can alter the sensitivity of the hypothalamic–pituitary–adrenal axis, leading to phenotypes that are resilient or vulnerable to stress later in life [16].

Human studies suggest that epigenetic regulation of gene expression plays a critical role in the long-term effects of early-life exposure to stressors [13]. DNA methylation, catalyzed by DNA methyltransferases (DNMTs), is one of the best understood epigenetic modification mechanisms [17]. The effects of early life stress, such as famine [18,19], infant-parental separation [20,21], and cold exposure [11], on long-term physiological changes are associated with DNA methylation status. Both early-life stress [22] and adulthood chronic stress [23] altered *CRF* expression through DNA methylation. We hypothesized that stressor-induced changes in DNA methylation lead to increased hypothalamic expression of *NPY* and *CRF* in LWS. Thus, the objective herein was to determine the effects of stress on DNA methylation in the ARC and PVN of LWS chicks and to elucidate the associated molecular mechanism.

## 2. Materials and Methods

### 2.1. Animals and Experimental Design

All animal protocols were approved by the Institutional Animal Care and Use Committee at Virginia Tech and animals were cared for in accordance with the National Research Council Publication, Guide for the Care and Use of Laboratory Animals. The LWS chicks were hatched at the Paul B. Siegel Poultry Research Center at Virginia Tech. Eggs were from age-contemporary breeders from the 61st generation. All experiments were done within one hatch. On the day of hatch, chicks were placed inside cardboard boxes (37 × 24 cm, n = 20 per box) and divided into stressed (S) and non-stressed (NS) groups. The boxes, experimental design, and stress protocol were the same as implemented in our previous stress studies [4,5]. The chicks in the stress group were subjected to −20 °C for 6 min and then transferred to 22 °C for 24 h without food or water provided. The control chicks were group caged at 32 ± 1 °C and 50 ± 5% relative humidity with free access to diet (21.5% crude protein and 3000 kcal ME/kg) and water. On day 1 post-hatch (24 h post-stress), all chicks were transferred to individual cages in which rearing conditions and food were the same as for the control group. Chicks had visual and auditory contact with each other in the individual cages and were handled twice daily to adapt to handling. All chicks were sexed by gonadal inspection post-euthanasia.

### 2.2. Body Composition

Fat and lean masses were measured (n = 6 males per group) with a minispec LF90 NMR whole body composition analyzer (Bruker, Billerica, MA, USA). The instrument was calibrated with a bottle of 500 g of canola seeds before use as recommended by the manufacturer. Each chick was scanned twice, and the average of the duplicates was used for data analysis. Prior to the measurements, yolk sacs were completely removed from the euthanized chicks by excision through the navel and weighed. This procedure was necessary to eliminate the confounding effects of residual yolk nutrients on whole body composition. Sex was determined by visual inspection of the gonads.

### 2.3. Nuclear Protein Extraction and DNMT Activity

On day 5 post-hatch, chicks were decapitated (head and neck) and then perfused via the carotid artery with hypotonic buffer (10 mM HEPES, pH 7.9, with 1.5 mM MgCl_2_ and 10 mM KCl). Brains were mounted with Tissue-Plus O.C.T compound (Fisher HealthCare, Houston, TX, USA), snap-frozen in liquid nitrogen, and then sectioned in the direction from rostral to caudal in a cryostat at −10 °C into 500 μm thick coronal sections within 30 min post-perfusion. The PVN and ARC were collected at 7.4 and 5.4 interaural, respectively, based on the Kuenzel and Masson chicken stereotaxic atlas [24]. Nuclei were collected using sterile disposable biopsy punch instruments (1 mm; Braintree Scientific Inc., Braintree, MA, USA) and were immediately transferred to sterile microcentrifuge tubes containing 25 μL pre-mixed pre-extraction buffer provided in the EpiQuik Nuclear Extraction Kit I (Epigentek, Farmingdale, NY, USA) and stored at –80 °C until further processing. To ensure anatomical accuracy, the remaining brain section was photographed and anatomy confirmed via digital overlays containing the respective nucleus boundaries according to the Kuenzel and Masson chicken stereotaxic atlas [24]. The nuclear extraction procedure was performed with the EpiQuik Nuclear Extraction Kit I (Epigentek), following the manufacturer’s protocol. Protein concentration was determined with a Bradford assay (Bio-Rad, Hercules, CA, USA). The EpiQuik DNMT Activity/Inhibition Assay Ultra Kit-Colorimetric (Epigentek) was used to determine DNMT activity, following the manufacturer’s protocol with 10 μg of each of the nuclear extracts. The absorbance (optical density; OD) was detected at 450 nm using a M200 Pro Multi-Mode plate reader (Tecan, Männedorf, Switzerland). Specific activity was calculated as

$$\mathrm{DNMT}\;\mathrm{activity}\;\left(\mathrm{OD}/h/\mathrm{mg}\right)=\frac{\mathrm{Sample}\;\mathrm{OD}-\mathrm{Blank}\;\mathrm{OD}}{10\;\mu g\;\mathrm{Protein}\;\mathrm{input}\times 2}\times 1000$$

Six to nine samples from each treatment group were used for statistical analysis after exclusion of those that exceeded a two-fold standard deviation (SD) from the average.

### 2.4. Genomic DNA Extraction

On day 5 post-hatch, chicks were decapitated (head and neck) and then perfused via the carotid artery with 1.5 mL of nucleic acid stabilizing buffer (16.7 mmol/L sodium citrate, 13.3 mmol/L EDTA and 3.5 mol/L (NH_4_)_2_SO_4_; pH 5.2). Brains were sectioned and the PVN and ARC were collected as described above. Collected nucleus samples were immediately transferred to sterile microcentrifuge tubes containing DNA lysis buffer (Norgen Biotek, Thorold, ON, Canada). Samples were vortexed, snap-frozen in liquid nitrogen, and stored at −80 °C until further processing. The genomic DNA was isolated according to the manufacturer’s instructions for the Cells and Tissue DNA Isolation Micro Kit (Norgen Biotek). The concentration and purity of total DNA was assessed with a Nanophotometer Pearl spectrophotometer (Implen, Westlake Village, CA, USA) at 260/280/230 nm. Genomic DNA quality was verified by 1.2% agarose gel electrophoresis. Six to seven genomic DNA samples from each group were used for global DNA methylation quantification, and six to eight genomic DNA samples from each group were used for bisulfite conversion and downstream sequencing after exclusion of those that exceeded a two-fold SD from the average.

### 2.5. Global DNA Methylation Quantification

Global DNA methylation was quantified with a MethylFlash Methylated DNA Quantification Kit-Colorimetric (Epigentek) following the manufacturer’s protocol. The input DNA amount was 100 ng per reaction. The absorbance was measured at 450 nm and the percentage of DNA methylation (5-methyl cytosine %; 5-*mC*%) was calculated according to the following equation:

$$5-mC\;\%=\frac{\left(\mathrm{Sample}\;\mathrm{OD}-\mathrm{Negative}\;\mathrm{control}\;\mathrm{OD}\right)÷\;100\;\mathrm{ng}\;\mathrm{DNA}\;\mathrm{input}}{\left(\mathrm{Positive}\;\mathrm{control}\;\mathrm{OD}-\mathrm{Negative}\;\mathrm{control}\;\mathrm{OD}\right)\times 2÷5\;\mathrm{ng}\;\mathrm{Positive}\;\mathrm{control}}$$

### 2.6. Bisulfite Conversion, PCR, and Sub-Cloning

Bisulfite conversion was performed with the Epitect Fast DNA Bisulfite Conversion Kit (Qiagen, Germantown, MD, USA) following the manufacturer’s instructions, with 100 ng of genomic DNA per reaction. Specific CpG-rich fragments in the promoter regions of *CRF* and *NPY* were amplified with EpiTaq HS (TaKaRa Bio Inc., Shiga, Japan). The primers were designed with MethPrimer [25] (Table 1). Each 50 μL PCR reaction contained 100 ng bisulfite-converted DNA, 1.25 U EpiTaq HS, 5 μL Mg^2+^-free 10× PCR buffer, 2.5 mM MgCl_2_, 0.3 mM dNTP, 0.4 µM each of sense and anti-sense primers, and nuclease-free water. PCR was performed with a Bio-Rad T100 Thermocycler (Bio-Rad) under the following conditions: initial denaturing step at 95 °C for 30 s followed by 40 cycles of 1) 95 °C for 20 s, 2) 51–61 °C (51 °C for *CRF* primers amplifying –288~35 bp upstream of the transcription start site (TSS), 61 °C for *CRF* primers amplifying –805~–645 upstream of TSS, and 52 °C for both sets of *NPY* primers) for 30 s, and 3) 72 °C for 30 s, and the final extension at 72 °C for 5 min. PCR products were resolved on 1.2% agarose gels from which the bands were excised and purified with a MinElute Gel Extraction Kit (Qiagen), following the manufacturer’s protocol. Purified PCR products were ligated into the pGEM-T easy vector system (Promega, Madison, WI, USA) at 3:1 or higher insert/vector ratio and incubated at 4 °C overnight. Ligated vectors were then transformed into JM109 competent cells (Promega) following the manufacturer’s protocol (including positive and negative controls) and plated onto LB plates with 100 μg/mL ampicillin (Sigma, St. Louis, MO, USA), 0.5 mM isopropylthio-β-galactoside (IPTG; Sigma), and 80 μg/mL 5-bromo-4-chloro-3-indolyl-β-*d*-galactopyranoside (X-Gal; Promega), and cultured at 37 °C overnight for blue-white screening. From each sample, six white colonies were amplified at 37 °C, 225× rpm, overnight, and plasmid DNA was extracted using a QIAprep Spin Miniprep Kit (Qiagen), following the manufacturer’s instructions. The concentration and purification of DNA were determined as described above. Prior to sequencing, target product sizes were verified for each sample by 1.2% agarose gel electrophoresis after EcoRI digestion.

### 2.7. DNA Sequencing and Sequence Analysis

Purified plasmid DNA samples were submitted to the Biocomplexity Institute at Virginia Tech for Sanger Sequencing. Sequences were viewed in 4Peaks (Nucleobytes, Aalsmeer, The Netherlands) and analyzed by Bisulfite Sequencing DNA Methylation Analysis (BISMA) software online. The analysis parameters were set as default with a lower threshold conversion rate of 95%, lower threshold sequence identity of 90%, upper threshold of N-sites at cytosine positions and upper threshold gaps of 20% allowed, and the detection of clonal molecules and alignment as suggested by the software. The methylation frequency of a single CpG site in each sample was determined from the six sequenced clones as the percentage of the 6 clones in which the site was methylated. The average of percent methylation was then determined for the group and the average used for statistical analysis. Overall methylation rate of a sample was calculated as (methylated CpG sites/all CpG sites in the amplicon) × 100%.

### 2.8. Chromatin Immunoprecipitation (ChIP) Assay

On day 5 post-hatch, chicks were decapitated (head and neck), and the whole hypothalamus was collected as described in Yi, Delp, Gilbert, Siegel, and Cline [5], except that the hypothalamus samples were directly snap-frozen in the liquid nitrogen instead of being submerged in RNA stabilizing buffer. Samples were quickly minced into small pieces on ice and cross-linked in 1% formaldehyde solution for 5 min, which was then halted with 0.125 M glycine. Tissues were further homogenized with a Kinematica Polytron PT 10/35 GT Homogenizer (Kinematica Inc., Bohemia, NY, USA) for 30 s and washed twice with ice-cold 1× phosphate-buffered saline (PBS; Hyclone, Logan, UT, USA), followed by a 10-min incubation on ice in cell lysis buffer (10 mM Tris-HCl (pH 8.0), 10 mM NaCl with 0.2% nonidet P-40 and 1× protease inhibitor cocktail (Thermo Scientific, Rockford, IL, USA)). Samples were then incubated in assay buffer (1% nonidet P-40, 0.5% sodium deoxycholate, 0.1% sodium dodecyl sulfate and 0.004% sodium azide in 1× PBS) on ice for 10 min, followed by sonication at 30 s on/30 s off for 30 cycles using a Bioruptor 300 with circulating chilled water system (Diagenode, Inc., Denville, NJ, USA). Then, 49 μL of sheared chromatin together with 1 μg of anti-methyl binding domain protein 2 (MBD2) antibody (Sigma) were used for each ChIP reaction using ChromaFlash One-Step ChIP kit (Epigentek) according to the manufacturer’s instructions. Two microliters of reverse-crosslinked DNA were added to each 20 μL PCR reaction containing 10 μL Fast SYBR Green Master Mix (Applied Biosystems, Carlsbad, CA, USA), 1 μL of 0.5 μM forward (5′-GGAGGCAGATTGCATACAGGA-3′) and reverse (5′-CCTCACAGAAGGCCCTAC-3′) strand primers and 6 μL nuclease-free water. The primers were designed with Primer Express, version 3.0 (Applied Biosystems) which amplifies *CRF* at –815~–631 bp upstream of the TSS. Reactions were performed under the conditions: 95 °C for 7 min followed by 40 cycles of 95 °C for 10 s and 60 °C for 30 s, and the final extension was at 72 °C for 1 min. Relative fold enrichment (FE) was calculated as $FE=2^{\left(Ct\;IgG-Ct\;Sample\right)}$, where IgG was used as the negative control in the ChIP assay.

### 2.9. Statistical Analysis

Body composition (except for fat mass), DNMT activity, global DNA methylation, and ChIP data were analyzed by *t*-tests and sequence data were analyzed by the Wilcoxon test using the Fit Y by X Model of JMP Pro 14 (SAS Institute, Cary, NC, USA). Because the stressed LWS had no fat in contrast to the non-stressed ones, these data were analyzed by Pearson’s chi-squared test in JMP Pro 14. Because preliminary statistical analysis with 2–3 replicates in each sex group and previous studies [4,5] showed no sex effect, treatment (non-stressed (NS) vs. stressed (S)) was the only effect included in the analyses. Differences were considered significant at *p* < 0.05. For all experiments, sample size information is provided in figures and reflects numbers after exclusion of outliers (greater than ± two standard deviations of the mean).

## 3. Results

### 3.1. Body Weight, Body Composition, and Yolk Sac Weight

**Figure 1:**
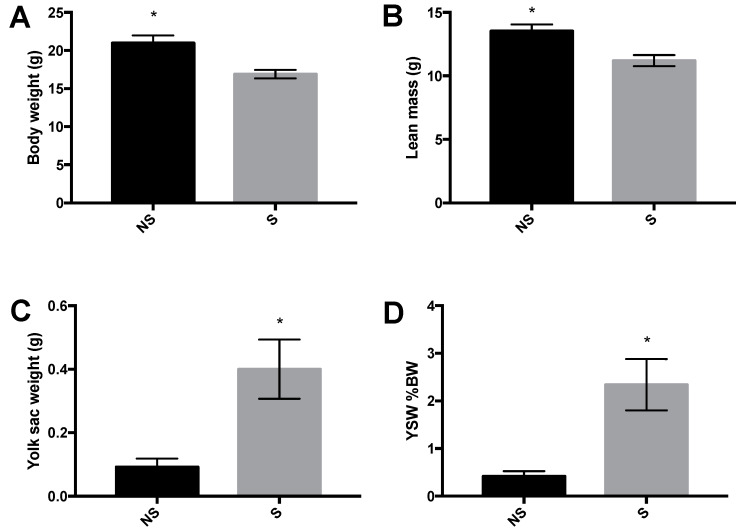
Body weight (**A**), lean mass (**B**), yolk sac weight (**C**), and yolk sac weight (YSW) on a body weight (BW) basis (**D**) of non-stressed (NS) and stressed (S) low body weight–selected (LWS) chicks on day 5 post-hatch. Data were analyzed by *t*-tests and are presented as means ± SEM. * *p* < 0.05, n = 6 per group.

Stressor-exposure was associated with reduced body weight (*p* = 0.008; Figure 1A) and lean mass (*p* = 0.007; Figure 1B) in LWS chicks at day 5 post-hatch. Stressor-exposed chicks had more yolk sac remaining than their non-stressed counterparts, both as an absolute weight (*p* = 0.02; Figure 1C) and as a percentage of body weight (*p* = 0.02; Figure 1D). Fat mass was not detectable in the stressed chicks at 5 days post-hatch, whereas the control birds had an average of 0.72 ± 0.15 g of fat (data not shown), accounting for 2.78 ± 0.56% of their body weight. Thus, stress affected fat deposition (*p* = 0.01).

### 3.2. DNMT Activity and Global DNA Methylation

**Figure 2:**
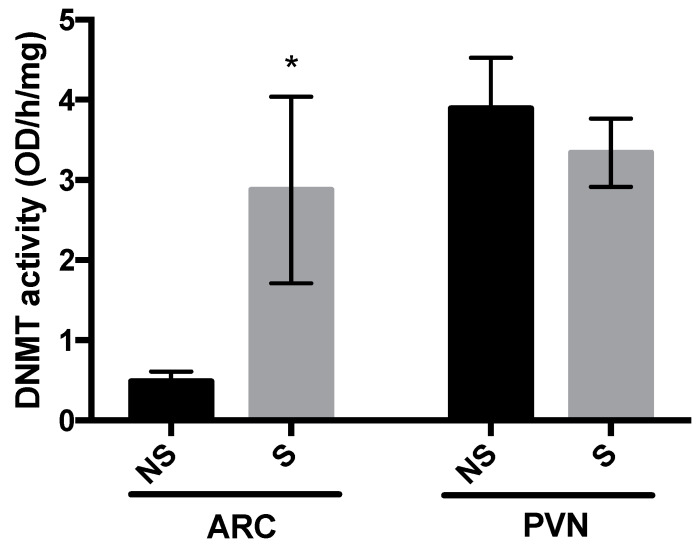
DNA methyltransferase (DNMT) activity in the arcuate nucleus (ARC) and paraventricular nucleus (PVN) of non-stressed (NS) and stressed (S) LWS chicks on day 5 post-hatch. Data (n = 7 for ARC NS, n = 6 for ARC S and n = 9 for PVN) were analyzed by t-tests and are presented as means ± SEM. * *p* < 0.05.

**Figure 3:**
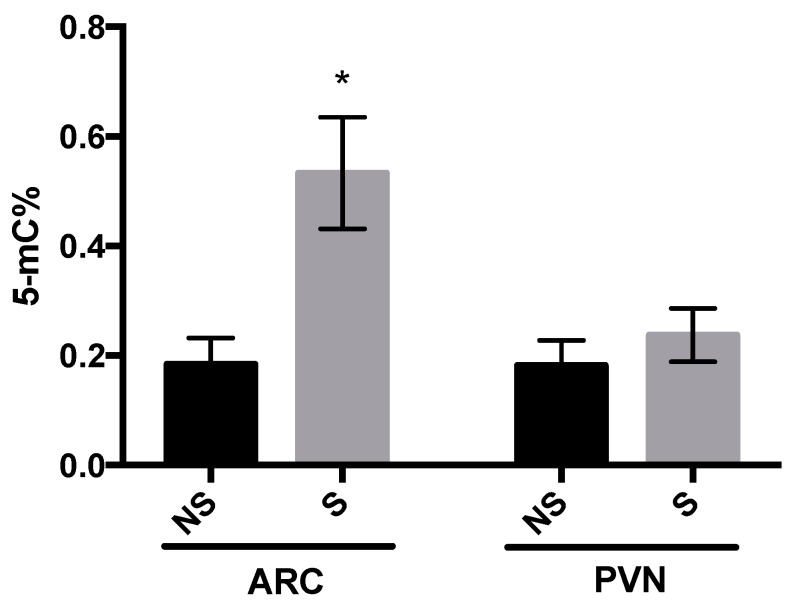
Global DNA methylation (5-methyl cytosine percentage; 5-mC%) in the arcuate nucleus (ARC) and paraventricular nucleus (PVN) of non-stressed (NS) and stressed (S) LWS chicks on day 5 post-hatch. Data (n = 6 for ARC NS and PVN S groups and n = 7 for ARC S and PVN NS groups) were analyzed by t-tests and are presented as means ± SEM. * *p* < 0.05.

In the ARC, DNMT activity was increased in stressed chicks at 5 days post-hatch (*p* = 0.04; Figure 2), whereas in the PVN, there was no difference in DNMT activity between the two groups. The same pattern was observed for global DNA methylation (Figure 3), where 5-methyl cytosine (5-mC) in the ARC was greater in stressed than non-stressed control chicks (*p* = 0.01), with no difference between groups in the PVN. Results here confirmed that stressor-exposure early post-hatch induced epigenetic changes in hypothalamus of LWS.

### 3.3. CpG Site Methylation at the CRF and NPY Gene

**Figure 4:**
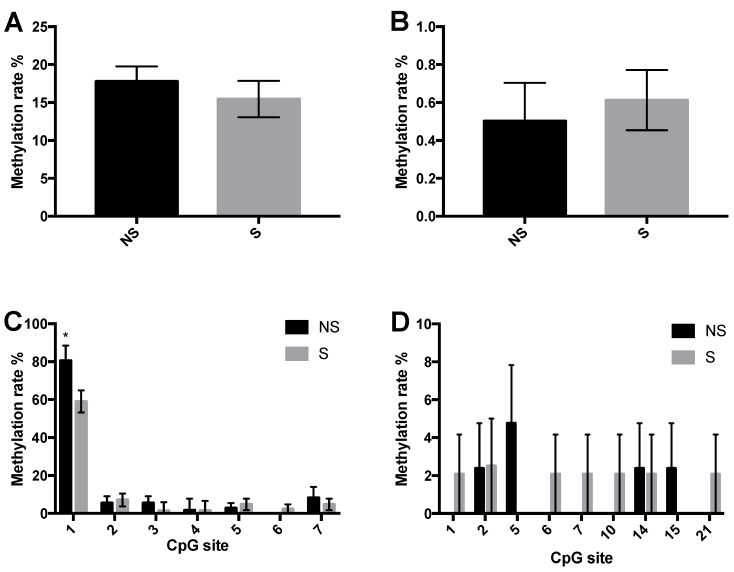
CpG site methylation status at the corticotropin-releasing factor (*CRF*) gene in the paraventricular nucleus (PVN) of non-stressed (NS) and stressed (S) LWS chicks on day 5 post-hatch. (**A**,**B**) Overall methylation frequency of seven CpG sites within –830~–670 bp upstream of the transcription start site (TSS) (5′→3′; A) and 24 CpG sites from upstream –288 to downstream 35 bp of the TSS (5′→3′; B) of the *CRF* gene. (**C**) Methylation rate of each CpG site within –830~–670 bp upstream of the TSS. (**D**) Methylation rate of each CpG site from upstream –288 to downstream 35 bp of the TSS (omitted sites in which no methylation was detected). Methylation rates in NS and S chicks (n = 7 for NS and n = 8 for S, except for Figure 4C, where n = 6 for NS and n = 7 for S) were analyzed by the Wilcoxon test and presented as means ± SEM. * *p* < 0.05.

**Figure 5:**
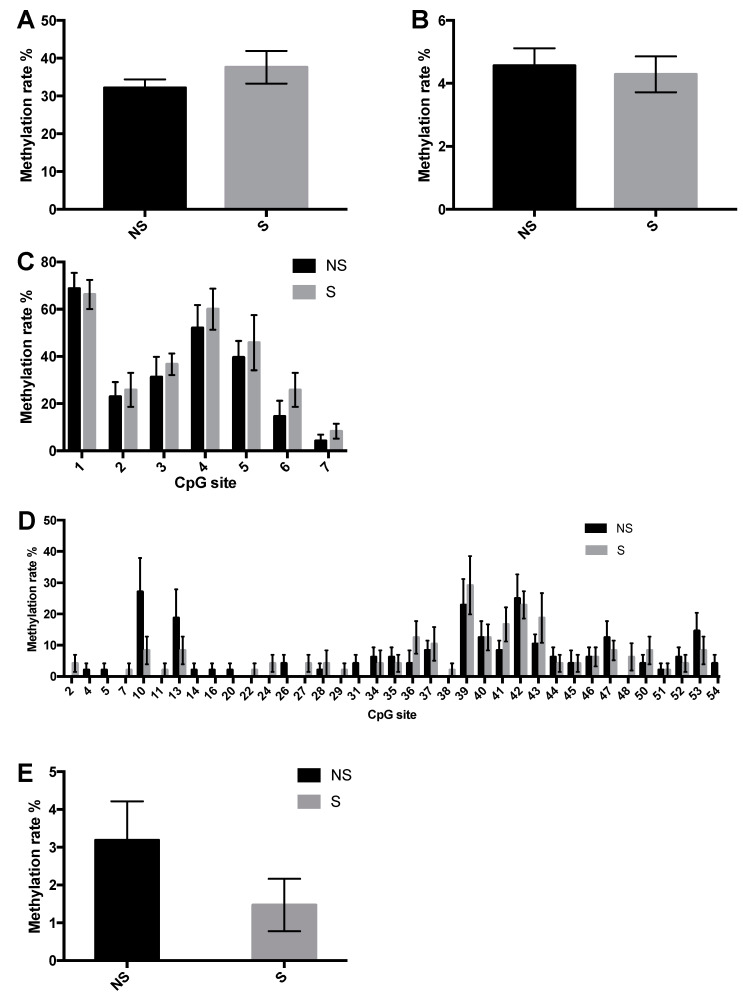
CpG site methylation status at the *NPY* gene in the arcuate nucleus (ARC) of non-stressed (NS) and stressed (S) LWS chicks on day 5 post-hatch. (**A**,**B**) Overall methylation frequency of 7 CpG sites within –594~–430 bp upstream of the transcription start site (TSS) (5′→3′; A) and 54 CpG sites from upstream –96 to downstream 416 bp of the TSS (5′→3′; B) of the *NPY* gene. (**C**) Methylation rate of each CpG site within –594~–430 bp upstream of the TSS. (**D**) Methylation rate of each CpG site from −96 upstream to 416 bp downstream of the TSS (omitted sites in which there was no methylation); (**E**) Methylation rate of CpG sites 4–20 at –96 upstream to 416 bp downstream of the TSS. Methylation rates in NS and S chicks (n = 8) were analyzed by the Wilcoxon test and are presented as means ± SEM.

To determine if previously observed mRNA changes in *CRF* and *NPY* in the stressed LWS are associated with DNA methylation changes near the respective genes, we calculated the methylation frequency of individual CpG sites near the CRF (Figure 4) and NPY (Figure 5) genes. One out of 31 CpG sites that were assessed in CRF differed (Figure 4C,D) where methylation was decreased in response to the stressors at 5 days post-hatch (*p* = 0.04). The overall methylation of all CpG sites in *CRF* and *NPY* was similar between groups (Figure 4A,B and Figure 5A,B, respectively), as was the methylation frequency of all 61 individual CpG sites that were sequenced on the *NPY* gene (Figure 5C,D). The overall methylation rate of CpG sites 4–20 from –96 upstream to 416 bp downstream of the TSS of the *NPY* gene approached significance (*p* = 0.0811), where methylation in stressed chicks tended to be lower than in non-stressed ones (Figure 5E). Thus, stressor-exposure at hatch changed DNA methylation status at the *CRF*, but not *NPY* gene promoter region. According to Scarsdale, Webb, Ginder, and Williams [26], this differentially methylated CpG site is within the core binding domain of methyl cytosine binding domain protein 2 (MBD2), a potent transcriptional repressor. Therefore, we next determined if binding to MBD2 is reduced in this region in response to stressor exposure.

### 3.4. Binding of MBD2 to the Promoter Region of CRF

**Figure 6:**
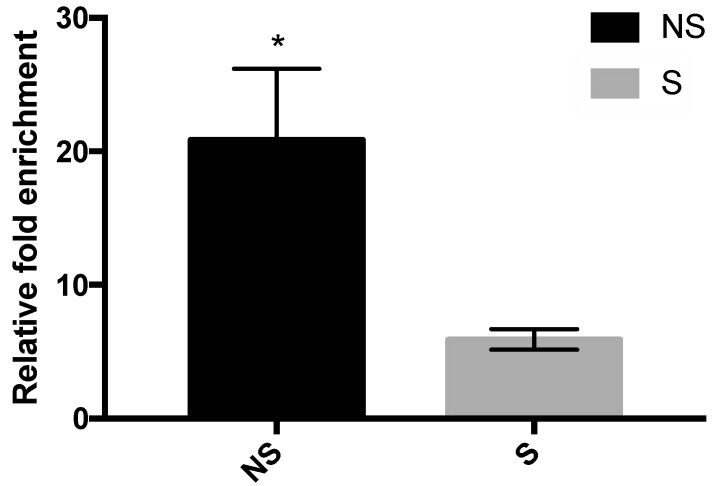
Relative fold enrichment of DNA recovered from anti-MBD2 antibody-bound chromatin in the hypothalamus of non-stressed (NS) and stressed (S) LWS chicks on day 5 post-hatch. Data (n = 10 for NS and n = 12 for S) were analyzed by *t*-tests and are presented as means ± SEM. * *p* < 0.05.

Relative fold enrichment of DNA reverse crosslinked from anti-MBD2 antibody-bound chromatin is shown in Figure 6. DNA enrichment was lower in the stressed than the non-stressed chicks (*p* = 0.02), indicating that in stressed LWS, binding to MBD2 at the promoter region of *CRF* is disrupted, relieving transcriptional repression, thereby leading to increased *CRF* expression.

## 4. Discussion

The hypothalamus has a critical role in integrating appetite and stress regulatory responses in order to maintain metabolic homeostasis. The LWS chicks that are exposed to a combination of nutritional and thermal stressors upon hatching are resistant to the orexigenic effects of centrally administered NPY five days later [5] and overriding anorexigenic tone originating from CRF production in the PVN plays an important role in the magnification of anorexia in these chicks [4]. We thus hypothesized that there was accelerated depletion of the yolk sac, the primary energy source for newly hatched chicks [27], upon exposure to stressors. However, at day 5 post-hatch, stressed chicks had greater amounts of yolk remaining (both on an absolute and relative to body weight basis) coupled to lower body weights than their non-stressed counterparts.

A similar phenomenon was observed in obesity-prone broiler chicks that were deprived of food immediately after hatching; they had more yolk remaining during the first three days post-hatch, and gained less weight [28]. In another study, chicks were housed in a cold environment (5 °C lower than optimal temperature) for five days with ad libitum or delayed access to food for 24 h right after hatch. Cold-exposed chicks showed no difference in growth from their control counterparts, whereas daily food intake and yolk resorption rates were greater, demonstrating that more energy is required to generate heat to maintain thermoneutrality. The chicks with 24 h food deprivation weighed less than the controls from day 2 to day 5 post-hatch. Although there was more potent yolk resorption immediately after the 24 h food deprivation, the remaining yolk mass was greater than in the controls at the end of the five-day trial. Without exogenous food, yolk is the only source of energy for survival, and its nutritional constituents are utilized efficiently through endocytosis (into the bloodstream). In the presence of food, however; more of the yolk is resorbed through the small intestine via the yolk stalk, demonstrating the importance of early-life nutrition to support optimal development of the gastrointestinal tract, where the presence of nutrients is an important stimulus for cellular growth [29]. In the present study, the combination of transient exposure to cold temperature and food deprivation likely induced a change in metabolism distinct from the effects of either stressor alone. This may explain why changes induced by thermal stress or nutritional deprivation in other studies produce distinct effects on appetite regulation, growth, and nutrient utilization.

Because the LWS chicks were exposed to cold with no access to food during the first 24 h post-hatch, and yolk utilization was not increased, we determined if mobilization of body reserves occurred to fulfill energy requirements for survival. As expected, stressed chicks had less lean mass and negligible amounts of fat. Exposure to a colder ambient temperature for three weeks post-hatch reduced broiler chick body weight gain, however body composition was not determined in that study [30]. In diet-induced obese mice, adulthood chronic stress decreased body fat mass and increased lean mass (both on a percent body weight basis) [31]. Although the effects of exposure to stressors at early ages on the alteration of nutrient metabolism during adulthood has been reported in rats [10], macaques [32] and mice [33], the effect on early neonatal body composition is unclear, especially in individuals predisposed to anorexia. To our knowledge, this is the first demonstration of effects of stress on early-life changes in body composition. Results herein indicate that increased mobilization of body reserves, instead of accelerated yolk resorption, may be the primary means of survival of stressor-exposed LWS chicks, which are inherently hypophagic and predisposed to anorexia. 

In our previous studies, there was up-regulation of *CRF* in the PVN and *NPY* in the ARC of stressor-exposed LWS chicks, and we postulated that CRF may override the orexigenic effect of NPY upon exposure to stressors and exacerbate anorexia in the LWS chicks at a later age [4,5]. In the present study, we determined whether such changes are related to stressor-induced alterations in DNA methylation patterns. There are more than 20,000 CpG islands in the chicken genome, and the global methylation rate of these islands is below 10% on average in different tissues of red jungle fowl and broilers [34]. This is in line with our observation that the amount of global DNA methylation in the ARC and PVN was below 1% regardless of treatment group. The exposure to stressors was associated with an increase in global methylation and DNMT activity in the ARC, but not the PVN. The assay for measuring DNMT activity does not distinguish among enzyme sub-types (de novo DNMT 3A and 3B vs. maintenance DNMT1). As global DNA methylation in a nucleus likely does not reflect the methylation changes at individual loci, we determined the DNA methylation patterns in CpG islands identified in the promoter region of *NPY* and *CRF*.

Although the critical role of CRF in mediating stress responses has been comprehensively reviewed [35], little is known regarding the mechanism through which *CRF* is upregulated by stressors. Females being stressed during early pregnancy resulted in de-methylation at specific CpG sites in the *CRF* promoter region in the hypothalamus and amygdala of the offspring, and this was associated with increased CRF expression in the amygdala [36]. Here we show that upon stressor exposure, there is reduced methylation status of a CpG site near the *CRF* promoter, in the PVN, that corresponds to the putative MBD2 binding domain. MBD2 is one of five methyl binding domain proteins found in mammals with each MBD protein binding to its preferred sequences [37]. It shows the greatest selectivity of methylated against unmethylated CpG sites, which facilitates more rapid and stable binding to methylated CpGs [38]. MBD2 is also found in chickens, sharing 83% sequence identity with human MBD2 [39]. The core binding sequence of chicken MBD2 is T(mC)GG [26], the same as the sequence containing the differentially methylated CpG in *CRF* in the PVN of stressed LWS chicks. DNA binding to MBD2 stabilizes the structure of the latter, which facilitates recruitment of the nucleosome remodeling and deacetylase co-repressor complex to repress transcription [40]. Thus, heavy methylation (~80%) of this CpG site in control chicks is likely associated with greater binding to the MBD2 protein, thereby permitting the recruitment of repressors to silence *CRF* expression under the non-stressed state. When LWS chicks were stressed immediately post-hatch, the methylation state of this CpG site was reduced substantially at day 5 post-hatch. We speculate that this is associated with reduced MBD2 recruitment, which results in an environment that is more permissible to transcriptional activation of *CRF* in order to modulate the downstream stress responses. This speculation is supported by our previous observations that *CRF* mRNA is up-regulated in the PVN of stressor-exposed LWS [4], which is coupled to increased circulating corticosterone [5]. The ChIP results further confirmed this speculation that binding of MBD2 in the promoter region of *CRF* was disrupted in the stressed chicks as a consequence of reduced methylation at a CpG site in its binding domain. That other CpG sites in *CRF* that we sequenced were all lowly methylated is consistent with the observation that methylation in the chicken *CRF* promoter region is low, and distinct regions of the *CRF* gene respond differently to stressors [16]. For instance, DNA methylation is enriched in the gene body, whereas gene expression is negatively correlated with methylation in the promoter region [34].

We also determined the methylation status of the *NPY* gene and our analysis covered 60 out of 78 CpG sites, close to the transcription start site (TSS) of *NPY* (both downstream and upstream), where there is high sequence conservation with the mammalian *NPY* genes and enrichment of predicted bindings sites for transcription factors [41]. Though not reaching statistical significance, the overall methylation rate of CpG sites at –46 upstream to 70 bp downstream of the TSS in *NPY* tended to be lower in the stressed chicks than the non-stressed ones. However, no methylation difference was detected for individual CpG sites sequenced in the *NPY* promoter region. Therefore, it is unclear if increased mRNA expression of *NPY* was related to changes in methylation within this region. In addition to its potent orexigenic effect, NPY also shows anti-stress effects in response to a variety of stressors (reviewed in Reichmann and Holzer [42]). Upon stressor exposure, CRF regulates the production of glucocorticoids, which are capable of upregulating the expression of NPY to alleviate the stress response and restore homeostasis [43]. Thus, upregulated *NPY* expression may not be directly modulated by methylation changes upon stressor exposure, but it could be a downstream target in the stress response. It is also possible that expression is regulated through other mechanisms, such as histone modifications or binding of transcriptional activators, or that other methylation sites not evaluated in this study were affected. Because we did not detect changes in methylation at the *NPY* gene nor evaluate other genes in the ARC, it is unclear whether the global hypermethylation observed in the ARC is related to changes in genes that inhibit appetite or induce satiety.

## 5. Conclusions

In conclusion, early post-hatch exposure to a combination of thermal and nutritional stress altered the utilization of yolk and impaired growth of the low body weight-selected chicks that are all hypophagic and predisposed to anorexia. Early post-hatch stress was associated with increased DNMT activity and global hypermethylation in the ARC but no change in the PVN. Results from gene-specific methylation analyses suggest that upregulation of *CRF* expression in the PVN of stressor-exposed chicks is associated with hypomethylation of a MBD2 binding site. Consequently, disrupted binding of transcriptional repressor MBD2 is likely associated with increased transcription of *CRF*. NPY may be a downstream effector of the stress response that is not directly modulated by DNA methylation. Our study provided further implications for understanding the molecular basis for eating disorders and identifying novel strategies to alleviate stress-induced changes in eating behavior.

## Figures and Tables

**Table 1:** Primers for Bisulfite Polymerase Chain Reaction (PCR)^1.^

Gene	Primer sequence (5′-3′); Forward/Reverse	Genomic Location	Amplicon Length (bp)	Number of CpG Sites	Location (Upstream of TSS)
*CRF*	AATCTCATTCAAATATTTTTA/ GAATTTGTGATTAGATTTGG	chr2: 115012110-115012433	324	24	−288~35
	GGATGTGTAATTTGAAGGAGGTAGA/ AACAAATCCCTCTAAAATCCCTTTA	chr2:115012790-115012952	161	7	−830~−670
*NPY*	AAAACACCATAAAACTATAA/ TTAGGAGAAGGGTAGTTTAG	chr2: 31464313-31464554	242	16	175~416
	ATAGTTTTTTAGAAGGTAGTTATGGG/ AATATCAAATCAATACCACAAACTC	chr2: 31463545-31463709	165	7	−594~−430
	GTTTAAGGTTTTTTTTGTTTGT/ ACCTCATAATACCCTACATCTAAAC	chr2: 31464042-31464328	287	38	−96~190

^1.^ Chicken corticotropin-releasing factor (*CRF*) and neuropeptide Y (*NPY*) gene sequences were analyzed with MethPrimer [25] and UCSC genome browser (https://genome.ucsc.edu/) to identify CpG islands in proximity to the promoter region. The location relative to the transcription start site (TSS) is indicated above.
